# Parent hospital experiences following stillbirth

**DOI:** 10.3389/fpsyt.2026.1706931

**Published:** 2026-02-04

**Authors:** Jennifer M. D. Kremkow, Jessica Lamberson

**Affiliations:** 1 Department of Communication Sciences and Disorders, Elmhurst University, Elmhurst, IL, United States; 2 Department of Nursing and Public Health, Elmhurst University, Elmhurst, IL, United States

**Keywords:** grief, parent experience, perinatal bereavement, stillbirth, survey

## Abstract

**Introduction:**

Stillbirth is an adverse pregnancy outcome, occurring in approximately 1:160 pregnancies that deeply impacts families and healthcare providers. Best practice guidelines have been established to provide a framework for bereavement care; however, there are still gaps in the provision of care to these parents.

**Methods:**

A qualitative research design in the format of a self-administered online survey was used. Purposive sampling was used to recruit participants in two phases–an open phase and a selected phase. Initial survey data were cleaned, resulting in 200 unique survey responses. The open-ended questions were analyzed using inductive content analysis.

**Results:**

The three main themes generated from the open-ended responses were Memory Making, Support, and Medical Decisions. Despite the majority of healthcare providers offering at least one memory making activity and the majority of parents participating in at least one memory making activity, the most common theme parents reported they would do differently was creating more memories.

**Discussion:**

Despite the current level of support provided by health care providers, potential gaps in bereavement care remain, indicating healthcare organizations and providers may intervene and improve bereavement care practices and parent experiences through consistent implementation and integration of perinatal bereavement care guidelines.

## Introduction

Stillbirth is a traumatic major life event experienced by parents anticipating a positive pregnancy outcome and occurs in approximately 1 in 160 births or 5.96 per 1,000 live births in the United States (1). Although there are varying international definitions of stillbirth, in the US, primary consensus defines stillbirth as fetal death after 20 weeks gestation. If gestational age is unknown, stillbirth is defined as fetal weight greater than or equal to 350 grams, excluding pregnancy terminations (1). Due to a lack of standardized protocols in evaluation and classification of stillbirth (1, 2), low rates of perinatal autopsy (1, 3), and associations of silence and stigma with stillbirth (4), evidence-based perinatal bereavement care (PBC) strategies remain inconsistent and insufficient (5).

Perinatal bereavement care encompasses a wide variety of care activities provided to bereaved parents and families following perinatal loss (miscarriage, stillbirth or neonatal death). PBC practices entail routine and specialized care, including, but not limited to diagnostic testing, meaning-making activities, grief support, and labor, birth, postpartum, and postmortem care. Many of these activities are initiated while a patient is in the hospital and are continued following hospital discharge. In the US, it is common that PBC activities are provided by an interdisciplinary team of health care providers.

A scoping review of international perinatal bereavement care guidelines in the immediate postpartum setting found key characteristics of PBC fell into five broad categories: memory making, good communication, effective emotional and social support, shared decision-making, and organizational support. While PBC characteristics were fairly consistent across settings, they were found to be implemented inconsistently (5). Additionally, there are few countries that have evidence-based national guidelines for perinatal bereavement care, including Australia and New Zealand, Ireland, and the United Kingdom (6–8).

Memory making allows parents the opportunity to spend time and create lasting mementos, such as photos or videos, with and in remembrance of their stillborn baby. It also allows for acknowledgement of the personhood of the baby, the parenthood of the parents, and the enduring relationship between them (9, 10). Good communication entails appropriate, respectful language and timely sharing of information as it relates to birth, postpartum, postmortem, and ongoing medical care following hospital discharge. Effective emotional and social support requires healthcare providers to understand and respond to varied grief responses and coordinate support services for short and long-term health needs. Shared decision making ensures that patients are provided the necessary information to make healthcare decisions regarding their care and the care of their stillborn baby, in conjunction with their healthcare providers. Organizational support implies a responsibility for institutions to maintain formal standardized policies and procedures to support PBC implementation and training for healthcare providers (1, 5–9, 11).

In the absence of national guidelines, high-quality clinical practice guidelines provide reliable evidence to guide the implementation of PBC by health care providers. Evidence-based PBC practices can also be integrated into training perinatal healthcare professionals, who, in turn, care for patients and families experiencing perinatal loss. Despite the development of evidence- and consensus-based PBC practice guidelines for health care professionals (1, 5, 6, 11, 12), their consistency, quality, and implementation varies (5, 11). An analysis of the methodological quality of eight published PBC clinical practice guidelines, revealed only two were “strongly recommended” for direct and immediate use (13). Additionally, health care providers cite feeling underprepared to provide adequate care to bereaved parents and needing formalized training in perinatal bereavement care (5, 11, 14–16).

Although PBC clinical guidelines exist, it is important to know if and how these guidelines are being implemented in practice. Understanding parents’ perceptions and experiences at the hospital while giving birth to a stillborn baby offers some insight into common current practices and gaps that may need to be addressed. Without this information, it is difficult to make meaningful changes in the provision of PBC for families experiencing a stillbirth. This paper sought to address this gap by surveying bereaved parents about their stillbirth hospital experience. Specifically, the main research question was: what is the experience of parents experiencing stillbirth while at the hospital?

## Methods

### Study design

The research team consisted of two researchers, each with a different background related to stillbirth, providing insider and outsider perspectives (17). The first author (JK) is a speech-language pathologist with research experience using a variety of designs, including qualitative methodologies. JK also has the lived experience of her second daughter being stillborn in 2023, which offers an insider lens to aid interpretation of participant experiences. The second author (JL) is a registered nurse with clinical experience as a certified inpatient obstetric nurse. She has cared for patients and families of stillborn babies and offers an insider lens to aid interpretation of health care providers, systems, and processes. JL also approaches the research from an outsider perspective, not having experienced a stillbirth.

A self-administered online survey was chosen to determine the parent-to-parent advice of parents experiencing stillbirth and neonatal loss. Online surveys allow researchers to understand the perspectives of a large number of participants across a broad geographic area within a relatively short amount of time (18). Although the online survey included parents experiencing stillbirth and neonatal loss, only stillbirth data related to the research questions were presented in this manuscript.

The content for the survey was developed through a review of literature related to stillbirth [e.g., (19)], neonatal death, [(e.g., (20)] and researcher personal experience. The final stillbirth version survey contained approximately 55 questions about the following categories: demographic information, advice parents would give other parents for the hospital, advice parents would give other parents for the grief journey, and preference for future studies (see **Supplementary Materials**). Data analysis for this manuscript included the demographic information and eight questions from the advice for the hospital sections of the survey.

Survey questions were created following best practice guidelines in survey question design [e.g., (18)]. Questions were written to be simple, straightforward, and ask about one topic at a time with responses presented in a logical order (e.g., alphabetical) and open-ended questions used cautiously and strategically. Additionally, questions were crafted for sensitivity and personalization. Pregnancy and infant loss may be a highly emotional topic for parents, requiring thoughtfulness in the language and terminology used. For example, respondents were given the option to write their child’s name in a free response question and see their child’s name throughout the remainder of the survey. If respondents granted permission, the baby’s names are used in this manuscript as a way to honor and remember them, something parents who have a stillborn baby often desire (19).

### Research governance

This study was approved by The Elmhurst University Institutional Review Board (IRB) on March 12, 2024 (ID FY024-036). The researchers were awarded a university grant for this project which was used for small participant incentives in the form of a raffle for a $5 gift card or a $5 donation to an organization of their choice. While there was no formal patient/public involvement in this study, both researchers have personal and/or professional experience in the area of stillbirth. Additionally, the first author utilized other parents with experience in stillbirth and/or neonatal loss in the development and piloting of this survey.

### Recruitment, data collection, and participants

Participants were recruited through a variety of techniques using purposive sampling procedures. Recruitment took place in two phases – an open phase and a selected phase. During the open phase, researchers distributed the study flyer through social media channels. Within hours of distributing the survey during the open phase, researchers noticed a high volume of low-quality responses. Researchers paused active recruitment but did not remove already distributed study fliers from social media channels. While active recruitment was paused, researchers reviewed additional literature on online surveys, recruitment, and bot detection and developed a second, targeted recruitment phase. During the selected phase, researchers removed the incentive from study flyers, created a new link to a slightly revised survey, and distributed the study flyer in four targeted ways to reach possible participants. First, the researcher with experience in stillbirth joined closed Facebook groups related to the topic (e.g., stillbirth parent groups) and received permission from moderators to post the study flyer. Second, the researcher with experience as a labor and delivery nurse contacted personal and professional contacts with experience related to the topic (e.g., labor and delivery nurses) to share the study flyer with those who may be interested. Third, researchers contacted professional organizations related to the topic (e.g., stillbirth non-profit organizations) and requested they share the study flyer. Finally, the researcher with experience in stillbirth contacted parents who formed local pregnancy and infant loss support groups and non-profit organizations and requested they share the study flyer. Active recruitment for the survey lasted for approximately six months.

After IRB approval, the researchers contacted a selected number of personal contacts with experience relevant to the research question to serve as a pilot test for the questions. Feedback from pilot respondents was incorporated into the final version of the survey. For example, some terminology was added (e.g., including miscarriage as an option for a pregnancy outcome), instructions for questions were updated (e.g., leave item blank if it does not apply), and questions were expanded (e.g., open ended question for helpful resources). The open phase of recruitment began after edits were made to the survey following pilot review. The selected phase of recruitment began after minor edits were made to the survey for enhanced validation and revising the recruitment strategy to be more targeted to the population. Individuals interested in participating in the survey accessed the Qualtrics survey link through study fliers and announcements. In the consent, individuals were informed of the study, its purpose, and their rights as a participant. If an individual consented to participate, they were provided access to the survey. Survey respondents were able to leave the survey at any time or could skip questions they preferred not to answer, unless questions were required for eligibility or validation. Survey responses collected were anonymous; however, if respondents wished to be eligible for a raffle for compensation and/or participate in future research, they were asked to provide their name and email address. After survey data were cleaned and a final set of accurate responses were determined, researchers randomly chose 120 respondents to receive or donate a $5 gift card.

Participants must have fulfilled the following inclusion criteria to be included in data analysis for this manuscript (1): be ≥18 years old (2); be the parent of a stillborn baby (≥20 weeks gestation or have a self-identified stillbirth experience near 20-week gestation) (3) have access to the internet (4), be fluent in written and verbal English (5), consent to participate in the study, and (6) answer at least 50% of the survey questions related to the research question. The inclusion criteria were verified via participant responses to demographic questions on the survey. At the time of the study, participants ranged in age from 20-65+ years with the majority of participants residing in the United States (84.5%) and identifying as White/Caucasian (79.5%), straight/heterosexual (94.5%), and the person who was pregnant while experiencing the loss (95%). The majority (81.5%) of participants had experienced multiple pregnancies with 38.5% of participants experiencing multiple pregnancy losses. The majority (61.1%) of stillbirths occurred between 33-40+ weeks gestation. All stillbirths occurred between 17-40+ weeks gestation. While the medical definition of stillbirth in the US is frequently 20+ weeks, the authors also included those who self-identified as having a stillbirth and reported an in-hospital experience of giving birth to a baby without a heartbeat. There were five participants who had losses ranging from 17–19 weeks gestation included in this data set. More detailed information about the demographics of participants is in **Table 1**.

**Table 1:** Parent demographic data.

Item	n	%
Age	200	
20–29 years old	30	15.0
30–39 years old	109	54.5
40–49 years old	54	27.0
>50 years old	7	3.5
Gender	200	
Female	187	94.0
Male	11	5.5
Non-binary	1	0.5
Did not answer	1	0.5
Race/Ethnicity	200	
American Indian, Alaska Native, First Nations	1	0.5
Asian, Asian American	5	2.5
Black, African American	10	5.0
Hispanic	8	4.0
Latino/a/e	1	0.5
Middle Eastern, North African, Arab American	1	0.5
White, Caucasian	159	79.5
A race or ethnicity not listed here^a^	5	2.5
Multiple Race/Ethnicity^b^	10	5.0
Sexual Orientation	200	
Bisexual	7	3.5
Gay or lesbian	1	0.5
Heterosexual or straight	189	94.5
Prefer not to answer	2	1.0
An orientation not listed here	1	0.5
Residing Country	200	
USA	169	84.5
Other^c^	31	15.5
Parent Loss Experience	200	
Person who was pregnant	190	95.0
Partner of someone who was pregnant	10	5.0
Gestational Age of First Stillbirth (weeks)	190	
17-24	39	20.5
25-32	35	18.4
33-40	109	57.4
>40	7	3.7

*n* varies per item 190–200 as indicated for each item.

a A race or ethnicity not listed here included Australian n=2, Irish n=1, Sri Lankan n=1, and French n=1.

b Multiple Race/Ethnicity respondents included Latino/a/e, Brazilian n=1; Black African, Portuguese n=1; Hispanic, White, Caucasian n=2; Black, African American, White, Caucasian n=2; Hispanic, Latino/a/e n=1; and Hispanic, Latino/a/e, White, Caucasian n=1.

c Other (residing countries with <10 respondents) were Canada n=8, UK n=7, Australia n=5, South Africa n=2, Denmark n=1, Germany n=1, Ireland n=1, Netherlands n=1, Northern Ireland n=1, Rwanda n=1, Uzbekistan n=1, United Arab Emirates n=1, and Venezuela n=1.

### Data analysis and reliability

Survey data from the open phase and the selected phase of recruitment were rigorously cleaned and analyzed to determine accuracy of each respondent. First, survey data were divided into categories to determine which responses would receive further analysis and which responses were insufficient for further analysis (e.g., did not consent, did not answer any demographic questions). Second, responses related to stillbirth were separated from those related to neonatal loss. Third, stillbirth data were analyzed to determine fraudulent responses related to duplicate responses in some items (i.e., start date/time, end date/time, IP address, latitude, longitude) and repetition in the first open ended question. Fourth, stillbirth data were analyzed to determine nonsensical or inconsistent answers to open ended questions. The second author confirmed analysis and authors reached 100% agreement. Any respondent flagged with at least one nonsensical or inconsistent answer was reviewed in full. Fifth, stillbirth data were analyzed for completion of demographic and relevant survey section (i.e., at least 50% of advice at the hospital questions completed). Any responses not meeting the requirement for demographics and survey section were coded out. Sixth, stillbirth data were checked for other inconsistencies (e.g., number of pregnancies, name of child, any patterns in matrices). Any respondent flagged with at least one inconsistency was reviewed in full. At each step, fraudulent responses were coded out of the data set. The remaining 200 responses were included in this analysis.

After a final set of accurate survey responses was determined, descriptive statistics were used to characterize the demographic questions and close-ended questions. The authors applied an inductive content analysis to the open-ended question, allowing the data to determine the coding labels during the analysis process (21, 22). Both authors familiarized themselves with the data and then analyzed all responses to the open-ended question to determine broad, “big-picture” categories or themes. The authors then met to compare and discuss themes, identify sub-categories, and generate operational definitions of the themes. The first and second author applied the operational definitions to approximately 52% of the data set to obtain inter-rater reliability. Inter-rater reliability was calculated by dividing the number of agreements by the total number of codes, then multiplying by 100 to obtain a percentage. Inter-rater reliability was 81% which is considered reliable and not likely to occur by chance (23). The first author coded the remainder of the data set.

## Results

Quantitative and qualitative analysis of seven questions related to what parent respondents experienced at the hospital generated three main themes: memory making, support, and medical decisions. Quantitative questions asked parent respondents to reflect on what healthcare providers did or offered that brought them comfort at the hospital, what information healthcare providers gave them related to stillbirth and grief, and what (if any) memory making activities they or their partners participated in at the hospital. The qualitative question asked parent respondents to reflect on what they would change or do differently at the hospital.

### Memory making

Parents were asked what type of memory making activities were provided by healthcare professionals while at the hospital for the birth of their stillborn baby (see **Table 2**). The majority of parents (95%) indicated healthcare professionals offered at least one type of memory making activity with their baby. The average number of memory making activities offered by healthcare providers was 4/6 options listed with a range of 0-6. The most commonly selected memory making activities offered by healthcare professionals were photographs, general encouragement to consider making memories, and hand and/or footprints.

**Table 2:** What did the healthcare professionals do or offer that was helpful or provided comfort for you while at the hospital?

Item	n	%
Making memories		
Took photographs	168	84.0
Encouraged memory-making	167	83.5
Created hand/footprints	163	81.5
Offered guidance	145	72.5
Clipped hair	86	43.0
Created hand/foot molds	98	49.0
Acknowledging parenthood		
Expressed condolences	185	92.5
Asked baby’s name	168	84.0
Said baby’s name	153	76.5
Complimented baby	146	73.0

*n* = 200.

Similarly, most parents (95%) indicated healthcare professionals acknowledged their parenthood in at least one way with expressing condolences being the most common. On average, parents reported healthcare professionals acknowledged their parenthood in 3/4 options listed with a range of 0-4.

Parents were asked if they participated in any memory making activities with their stillborn baby while at the hospital and if so, what memory making activities did. Parents were also asked to report any memory making activities their partners did with their baby (see **Table 3**). In total, 88.9% of parents reported making memories with their baby at the hospital. Out of the 177 parents who reported making memories, 174 parents also completed a table about specific memory activities they or their partner participated in while at the hospital. On average, parents listed 6/11 memory making activities either they or their partner did at the hospital with a range of 0-11. The most common memory making activities were spending time with their baby (97.7%), taking photos or asking for photos of their baby (87.9%), naming their baby (87.4%), talking to their baby (83.3%), and creating or asking for hand or footprints of their baby (63.2%).

**Table 3:** What memory making activities did you or your partner do at the hospital?

Item	Parent respondent	Partner of respondent	% One or both parents
Spend time with your baby	164	149	97.7
Take photos/ask for photos of your baby	145	129	87.9
Name your baby	146	132	87.4
Talk to your baby	142	116	83.3
Create/ask for hand or footprints	97	66	63.2
Dress your baby	57	46	40.2
Create/ask for hand or foot molds	55	37	34.5
Sing to your baby	47	29	29.9
Clip/ask for clippings of hair	40	26	27.6
Bathe your baby	24	22	17.8
Read to your baby	20	20	14.4
Other	NA	NA	23.0

n = 174.

In addition to the 11 memory activities listed as options on the survey, 40 respondents chose to indicate “other” and further describe the memory making activities with their baby. Some parents commented more than once for a total of 54 responses. “Other” memory making activities were categorized into five groups (see Appendix A). The most commonly mentioned memory activity that parents added on their own was related to physical contact (n=18) like holding, sleeping, or cuddling with their baby.

“Memory Making” for the qualitative question was defined as comments related to memory making activities or spending time with their baby that parents wished they had done differently (see **Table 4**). Nearly three quarters of parents (73.8%) indicated they wished they would have made more memories with their baby at the hospital. Sometimes parents expressed one specific memory making activity they would have done, such as having physical contact with their baby (e.g., holding) or keeping a physical memento (e.g., ashes). Other parents expressed one specific memory making activity they would have done more, which was often taking more pictures or videos or spending more time with their baby. Benjamin’s mother expressed the intensity of this desire by stating,

**Table 4:** Summary of themes and subthemes for what parents experiencing a stillbirth would change or do differently while at the hospital.

Theme	Subtheme	% (n)	Examples
Memory Making		73.8% (137)	
	Photo/Video	34.8% (65)	“I deeply regret not taking photos.” - Jack’s mother “I think we might have taken even more photographs. I felt a little awkward about ‘posing’ for photographs, but there was no need. It is completely normal to need to photographs afterwards, and this is so much of what you have - do everything you can.” - Aubrey’s father
	Physical Contact	25.1% (47)	“I wish I would have done skin to skin as soon as she was born” - Charlotte’s mother “I wish I held or cuddled with him more” - Shea’s father “Touch and hold your baby as much as you can, I spent a lot of time just looking at her in the cuddle cot but I wish I would have held her more.” - Elliana’s mother
	Time/Memories	24.6% (46)	“It’s one of my greatest regrets. I took the mindset of he was already gone, this was just his body. I did that to I think protect myself from what was happening. I wish I spent more time with him, holding him, and seeing him unswaddled.” - Thomas’ mother “I would have spent more than a few minutes with her.” - Jamie’s mother “I’d have just wanted more time with them.”
	Parenting	19.3% (36)	“I wish I dressed him. I was too scared to break him, though looking back that makes no sense.” - Cayden’s mother “Read her books, bathed her, changed her” - Tess’ mother
	Physical Memento	17.6% (33)	“I would have asked for the blanket she was wrapped in so I could bring it home.” - Lena’s mother “Get a clipping of her hair” - Jovie’s mother “I would have kept a small amount of ashes.” - JT’s mother
	Family	9.1% (17)	“I also would have had my living son come and see him. It felt like an impossible decision and I didn’t want to traumatize him so we decided against it. Looking back I think it would have been nice.” - Arlo’s mother “I wish I had let my other kids meet her.” - Levi’s mother
	See/View	8.0% (15)	“I wish I would have examined her body more, looking for freckles, birth mark, any type of unique features and take photos to remember.” - Elliana’s mother
Support		32.6% (61)	
	Information	18.7% (35)	“I wish I had been given more information about what we could do with Cricket.” - Cricket’s mother “Before [birth] I would’ve asked more questions or tried to mentally prepare myself more. I was still in denial until he was born.” - Liam’s mother “I do wish I’d had more information provided between learning my baby was gone and giving birth. I remember being absolutely shocked that I had to give birth so soon after learning after the loss. That shock carried through my entire experience.”
	Healthcare environment	16.6% (31)	“I wish the cuddle cot was offered. I wish we were encouraged to spend time.” - Ollie’s mother “I do wish at the hospital, someone had told me about Now I Lay Me Down to Sleep and I wish I got the chance to reach out for professional photos.” - Cayden’s mother “I was placed in a room next to a room where a living baby was, and hearing the baby all night was nightmarish.” - Honour’s mother
Medical Decisions		16.6% (31)	
	Maternal	10.7% (20)	“I would’ve documented my pregnancy journey better if I had known she would be stillborn.” - Lily’s mother “I would have gone sooner to the hospital as soon as I woke up and didn’t feel any kicks.” - Lilliana’s mother
	Baby	6.4% (12)	“I wish I would’ve done the autopsy” - Callie’s mother “My biggest thing is I wish we would have done an autopsy, it’s a very difficult thing to decide in the moment, but if an autopsy is a possibility for you to do it so you can hopefully find answers as to why this happened.” - Elliana’s mother “I think I may have opted to cremate, I agreed to a hospital cremation where we did not get remains.”

n = 187.

*“If I could relive one day, knowing it was one of the toughest of my life, I would do it to create more memories and preserve the time that we had with our son.”*

A majority of parents (55.8%) described at least two memory making activities they would have done differently. These activities were usually provided in a list with varying levels of detail. For example, Jackson’s mother stated,

“I wish I would have done more like change his diaper, read to him, more skin to skin, dress him in one of his many outfits we had bought him, taken more pictures of him.”

Elaina’s mother commented,

“I wish I would have asked for clippings of her hair, more molds (having just one makes me nervous to lose it). I wish I would have opened her eyes for the photographs or at least looked into them more. I wish I would have bathed her. I wish I would have brought clothes to put on her that I had gotten her. I wish I would have brushed her hair. I wish I would have had her sisters come hold her and more of my family meet her…”

One quote summarizing many of these activities expressed the desire for doing more memory making activities, but also suggested appearance and fear as possible barriers for parents while creating memories:

“I wish I spent more time with my baby. I wish I sang to her. I felt rushed because her appearance was changing fast. I wish I took more videos. I wish I asked to be the first one to hold her when she was born. I was scared.”

Several other parents echoed this sentiment in their description of memory making activities as well. For example, Finn’s mother stated,

“I would have seen him straight away after he was delivered rather than waiting until the next day. And I wish I had bathed and dressed him. I wish I hadn’t been so frightened to see him.”

Additionally, 91% of the 22 parents who indicated they did not make memories at the hospital explicitly stated they would have changed this in their open-ended response. These responses generally fell into two categories – specific memory making activities parents would have done or support they wished they had. Parents who indicated they did not make memories, but wish they had, mentioned memory making activities from nearly every category; however, most frequently listed were photos and physical contact. Madison’s mother’s response seemed to emphasize the importance of making memories:

“I never held her and I will live with that regret the rest of my life. I was so scared.”

Parents who indicated they did not make memories, but wished they had more information, commented on how they wished they knew they could make memories, but “[were not] offered anything”, were not sure what to do (“…I would have liked to know everything that I could do to make memories and then decide…”), or were not presented with memory making in a way that was helpful (“They said, ‘Hold him if you want.’ I didn’t like that verbiage” – Ollie’s mother). Abigail’s mother stated, she would do “everything” differently because, at the time, she didn’t know they could make memories with their daughter and no one presented this information to her.

### Support and information

Nearly all parents (97%) indicated healthcare professionals provided support in at least one other way during the hospital stay (see **Table 5**). On average, parents reported healthcare professionals provided support in 5/9 options listed with a range of 0-9. Providing pregnancy loss and/or grief resources was listed as the most common. In addition to the care measures or activities provided by healthcare professionals listed as options on the survey, parents had the opportunity to choose “other” and further describe support they received at the hospital. Forty-eight parents chose to provide additional comments in the “other” section, some of whom entered multiple comments for a total of 77 comments. “Other” comments from parents were categorized into five themes (see Appendix B). The most commonly mentioned type of support that parents added on their own was related to tangible support (n=35) like offering or creating a memory box, offering a cooling mat, or providing specific items such as a stuffed animal or blanket.

**Table 5:** What did the healthcare professionals do or offer that was helpful or provided comfort for you while at the hospital?

Item	n	%
Providing support		
Provided pregnancy loss/grief resources	166	83.0
Provided funeral/cremation services information	135	67.5
Said what happened was not parent’s fault	125	62.5
Recommended counseling/therapy services	120	60.0
Attached door signage indicating loss	116	58.0
Provided religious personnel/resources	111	55.5
Provided information on baby’s appearance	91	45.5
Located room separate from area with live births	88	44.0
Provided social work services	86	43.0
Other	48	24.0

*n* = 200.

Parents were asked questions related to the support and resources they received before or after the birth of their stillborn baby (see **Table 6**). The majority of parents (67.7%) reported not having support from someone who had also experienced a stillbirth while at the hospital. Similarly, the majority of parents reported not receiving any education materials from healthcare professionals about stillbirth (80.6%) or about grief related to pregnancy or infant loss (73.8%) before the birth of their stillborn baby. It was only after the baby was born that the majority of parents (74.1%) reported receiving this type of support.

**Table 6:** Support and resources parents received before and after birth of their first baby who was stillborn.

Item	n	%
While at the hospital, did you have support from someone who had experienced a loss similar to yours?	195	
Yes, a personal connection	23	11.8
Yes, a healthcare professional	29	14.9
Yes, an organization	11	5.6
No, I did not have support from someone who had experienced this type of loss	132	67.7
Before your baby was born, did you receive any education materials from healthcare professionals about stillbirth?	191	
Yes	17	8.9
No	154	80.6
I do not remember	20	10.5
Before your baby was born, did you receive any education materials from healthcare professionals about grief related to pregnancy or infant loss?	195	
Yes	32	16.4
No	144	73.8
I do not remember	19	9.7
After your baby was born, did you receive any educational materials from healthcare professionals about grief related to pregnancy or infancy loss?	193	
Yes	143	74.1
No	38	19.7
I do not remember	12	6.2

*n* varies per item 191–200 as indicated for each item.

For the qualitative responses, support was defined as comments related to the asking or offering of support desired before, during, or after the birth of the baby, including a lack of support (see **Table 4**). Support was the second most common theme parents would change while at the hospital (32.6%). Comments coded as informational support generally fell into two main categories. First, parents wished they had more information about the process of giving birth to a stillborn baby. For example, Gus’ mother commented,

“I didn’t have any preparation for what his birth would be like.”

Jules’ father said,

“I wish I had better understood what was going to happen, so I could be better prepared.”

Second, parents wished they had more information about memory making. Parents frequently stated they wished they would have done more memory making activities, but they did not know they could and no one offered them this information. For example, one parent said,

“I wish I had bathed my baby and I wish I had read to him. It didn’t occur to me and no one offered it. I also wish I had made a video of us with him, not just still pictures.”

Some parents commented on the emotional state they were in and how they wished someone would have spoken to them about making memories. Ida’s mother specifically commented on more information about stillbirth before the birth because,

“The nurses did some things, but after leaving the hospital we went home and found so many more resources and discovered things we would have loved to do with Ida, but didn’t consider because no one told us about it…”

Izzy’s mother offered a list of memory making activities she would have done differently and then commented,

“But I also give myself grace because I was quite literally in shock and had checked out mentally. But wish others would have taken more photos and gently encouraged me to make some memories with her. You NEVER imagine this happening. It’s not something most people consider or plan for. I had no idea what to do.”

Comments coded as environmental support were related to specific equipment, environment, or personnel changes parents would have made while at the hospital. Parents who mentioned equipment discussed the desire for a cooling mat to use for their baby. Noah’s mother said,

“I wish I asked for a Cuddle Cot and kept him in my room.”

Parents who commented about the environment were often describing minimizing exposure to other pregnant women or new babies while at the hospital. For example, Dylan’s mother said that she would have liked to not “go past all expecting mothers in the waiting room when they wheeled me into the maternity ward.” Parents who cited personnel often wished they would have had access more specialized personnel while at the hospital. Sometimes these personnel were related to memory making, such as professional photographers or organizations who photograph stillborn. Other times, these personnel were related to healthcare professionals or community members with specific training or experience in bereavement. For example, one parent stated:

“I wish I would have spent more time with her and did more to make memories. I wish I would have realized it was ok even if it felt weird/odd … I was scared/felt weird to do stuff like sing, talk to her, etc. … This is where I feel a bereavement doula would have been helpful.”

### Medical decisions

The third theme was smaller than the first two and only included data from the qualitative question. Medical Decisions was defined as comments related to choices parents wished they had done differently during their pregnancy or about the baby after birth (see **Table 4**). Maternal medical decisions included things parents wished they did differently during the pregnancy, such as taking more pictures while pregnant or telling more people about the pregnancy, advocating for themselves more, or getting more testing. Other parents commented on what they would have done differently related to maternal medical decisions while at the hospital during the birthing process such as pain management (“I wish I would have had an epidural to reduce the physical pain leading up to the worst emotional pain I have ever felt in my life” – Hugo’s mother). Baby medical decisions were centered around getting an autopsy or other testing and memorializing the baby. Emma’s mother described their rationale for wishing they had done an autopsy by emphasizing the importance for research and for peace of mind for future pregnancies:

“I wish I had the option of an autopsy presented to me differently. No one wants to think of an autopsy on their baby. But I had no idea at the time how vital data is to research and prevention even if it never gave us conclusive results of why she died. I wish instead of “It might not give you answers,” it had [been] shared that it would at least rule some things out. In our subsequent pregnancy we were scared of everything. Even knowing it wasn’t her heart would have been a comfort.”

## Discussion

This study surveyed 200 parents who experienced a stillbirth about their experience in the hospital, including support provided by healthcare providers, memory making activities, and anything they would do differently. Despite the current level of support provided by health care professionals, results from this study point to potential gaps in bereavement care indicating areas where healthcare providers may intervene. Given the significant impact healthcare providers have on the hospital experience of parents when their baby is stillborn (19, 24), addressing these gaps is critical to providing comprehensive care.

### Interpretation of findings compared to literature

Most of the parents in this study (95%) reported healthcare professionals offered or encouraged memory making in at least one way and reported making memories with their baby (88.9%). Many of the memory making activities discussed by parents in this study such as spending time with their baby, taking/asking for photos, and naming their baby, are commonly reported in bereavement literature (10, 25). Despite the majority of healthcare providers offering at least one memory making activity and the majority of parents participating in at least one memory making activity, creating more memories was the most common theme parents reported they would do differently. In fact, of the 22 parents who did not make memories at the hospital, 91% commented on how making memories is something they would change looking back. Clearly, the parents in this survey, as with parents in other studies [e.g., (10, 15, 19, 26, 27)], placed an emphasis on making memories as part of the hospital experience and have expressed regret at not making memories or wished they would have made more memories (28, 29).

Although memory making is regarded as an integral practice in national perinatal bereavement care guidelines (6–8), the results from this study indicated health care professionals may need to provide additional resources or guidance on making memories. Additional resources or guidance should carefully address some of the barriers to making memories described by parents in this study and others like information on what types of memory making activities are available (30) and normalizing the feelings around memory making (25, 31, 32). For example, it may be helpful for healthcare providers to have informational materials for the parents about what memory making is, what options for memory making are, and why other loss parents view memory making as important. To help normalize memory making, providers may emphasize how common it is that parents make memories with their baby (25) and validate emotions the parents may be feeling about making memories or the experience of stillbirth (10, 31).

Additionally, some parents in this study described the fear of their baby’s appearance as a barrier to memory making, but only 45.5% of parents reported healthcare professionals providing information on the baby’s appearance. While this is a difficult conversation to have, some parents want communication about their baby’s appearance after birth (19, 28). Healthcare providers should consider addressing this topic as part of the information they provide to parents before the birth of their stillborn baby by asking if the parents know what to expect after delivery and if they want this information. If the parents want to know, healthcare providers could gently provide information about their baby’s initial appearance and how it will change.

The majority of parents in this study reported healthcare professionals providing information related to pregnancy loss and grief (83.0%) with most information being provided after the birth of their baby (74.1%). Further, a majority of parents (67.7%) also reported not receiving support from someone who had experienced a stillbirth. However, a common theme that parents would change about their hospital experience was related to information, including the processes of giving birth to a stillborn baby and the postpartum period. Parents in other studies have been surprised by postpartum changes like lactation (24). Research shows that sensitive and timely communication of relevant information about the birthing process from healthcare providers is helpful and important to bereaved parents (15, 19, 27). Providing information about the birthing process, stillbirth, and grief before the birth of the baby, if possible, such as following the medical appointment where parents learn their baby does not have a heartbeat or after arriving at the hospital, may help prepare parents for the birth as well as making memories after, an opportunity they only get once. Healthcare providers could also partner with local or national loss organizations to connect parents to a network of individuals who have experience with stillbirth and can provide guidance to parents while in the hospital or help them through their grief journey after returning home. Previous literature indicated parents have appreciated connections hospitals had with external support groups and that these support groups are helpful (19, 27, 30).

Some parents reported healthcare providers adapting the environment to support their time in the hospital such as attaching a sign to the door indicating a loss (58.0%) or locating the parents’ room in an area separate from live births (44.0%). In open ended responses, some parents in this study mentioned seeing other pregnant women or hearing living babies while they were experiencing the birth of their stillborn baby is something they would change about their experience. Adapting the environment is likely not feasible in every situation; however, parents appreciated when care was taken to minimize environmental exposures to pregnant women and living babies (19) and described these environmental exposures as “[intensifying] their traumatic experience” when they were not minimized (24, p.5). Hospitals should consider flexible policies to allow adapting the environment when possible (e.g., sign on the door, room away from active live births, minimize contact with other pregnant women). When environmental adaptation is not possible, it may be helpful to inform the parents in advance to reduce the shock or anxiety of seeing another pregnant woman or hearing a living baby.

A few parents mentioned a cooling mat was another support healthcare providers offered that was helpful or provided comfort. Several parents also commented that having a cooling mat for their baby to use as something they would change about their hospital experience. The idea of having a cooling mat was often tied to the ability to make more memories with their baby. Hospitals should consider cooling mats or other equipment, practice, and policy supports to increase the time parents may have with their babies for making memories.

A small number of parents (5) specifically mentioned having a team of trained professional support like a bereavement nurse or doula who helped support them. Since most parents in this study did not have the support of someone who has experienced a stillbirth and parents have indicated that healthcare professionals left a lasting impression on them during a stillbirth experience in either positive or negative ways (28), working with a trained healthcare professional who can provide bereavement guidance becomes more important. Healthcare providers cite feeling unprepared and needing more training to provide perinatal bereavement care (11, 15, 33). Hospitals should consider providing or encouraging perinatal bereavement care training integrating clinical practice guidelines to educate healthcare workers who care for parents experiencing perinatal loss (6–8, 34–36). This training could support healthcare providers in facilitating difficult discussions around stillbirth, providing parents with information about their baby’s appearance, memory making options, autopsy decisions, partnering with external stillbirth organizations, and connecting with additional resources. Other options for providing additional bereavement support include partnering with local or national organizations supporting stillbirth parents or having a list of resources available for parents while they are in the hospital.

A few parents in this study mentioned they would change medical decisions related to their baby, including an autopsy and options for memorializing. Parents who wished they had done an autopsy indicated a need to understand the reasons why it might be helpful (e.g., future pregnancy), but to have it presented carefully because it was a difficult thing to think about. While many parents may receive information about autopsy, research indicates that various factors influence parents’ decision to proceed with postmortem evaluation. Parent perception of how postmortem information is presented, fear of further abandoning or harming their baby, dependence on others to make decisions during a highly-emotional time, and provider recommendations can impact parents’ decision to consent to an autopsy (15, 25, 26, 28). Healthcare professionals should approach the discussion of autopsy with sensitivity, recognizing the weight of their professional advice, while discussing potential benefits and acknowledging conflicting emotions parents may be experiencing.

### Strengths, limitations, and future directions

A strength of the study was the insider and outsider lens of the research team - one with an insider lens for the parent experience and one with an insider lens for the healthcare professionals - which enabled a multi-dimensional view of the data. Although a small percentage of yearly stillbirths within the US and worldwide, another strength of this study was the sample size (N = 200) which provided a range of rich experiences in close- and open-ended survey questions, some comments several sentences to paragraphs in length. While the data from our sample of 200 was descriptive, there are limitations related to the sample demographics that limit the broad application of the results. The parents who responded to this survey were overwhelmingly from the US and identified as White/Caucasian, straight/heterosexual, and female. It is likely that parents from outside of the US may have different experiences with stillbirth that were eclipsed in this dataset. Anecdotally, the only parents to indicate they did not receive any support from healthcare professionals during their time at the hospital were respondents from outside of the US. Parents from underrepresented racial and/or ethnic backgrounds or sexual orientation in this study may have different experiences in the healthcare system, including experiences of stillbirth [e.g., (37)]. Previous research has indicated fathers are underrepresented in the stillbirth literature, but may have different perspectives on their experiences than their partners [e.g., (38, 39)]. Similarly, people who identify as part of the LQGTQ+ community may experience different levels of stigma than heterosexual couples after a stillbirth (40). Future research should seek to address these limitations by investigating the stillbirth experiences of parents from various countries and demographic groups.

## Conclusion

This study provides some insight into the hospital experiences of parents experiencing a stillbirth, including what they would do differently if they had the opportunity. Even though best practice guidelines have been established to provide a framework for bereavement care [e.g., (6–8, 12)], our results suggest gaps in care still remain. Nearly all of the parents in this study described something they would do differently while at the hospital, despite most reporting a variety of support provided by healthcare professionals and participating in memory making activities. The type and timing of the information provided to parents experiencing a stillbirth by healthcare professionals may not address all of the parents’ needs while at the hospital. Healthcare organizations can improve institutional support to address these gaps in care by providing perinatal bereavement care training for interdisciplinary healthcare professionals. Additionally, healthcare providers who care for bereaved parents and families can consistently implement evidence-based perinatal bereavement care guidelines to improve the care and experience parents have during an unexpected and life-changing experience.

## Data Availability

The raw data supporting the conclusions of this article will be made available by the authors, without undue reservation.
