# Supplementary material for: Parent hospital experiences following stillbirth
Source: Front Psychiatry. 2026 Feb 4;17:1706931. doi: 10.3389/fpsyt.2026.1706931 (PMC12914944; doi:10.3389/fpsyt.2026.1706931)
Supplement: Supplementary file 1 [file DataSheet1.pdf]

Supplementary Materials – Selected Survey Questions

What is your age?

- ○ under 18 years old
- ○ 19 years old
- ○ 20-24 years old
- ○ 25-29 years old
- ○ 30-34 years old
- ○ 35-39 years old
- ○ 40-44 years old
- ○ 45-49 years old
- ○ 50-54 years old
- ○ 55-59 years old
- ○ 60-64 years old
- ○ 65+ years old

What is your gender?

- ○ Female
- ○ Male
- ○ Non-binary
- ○ A gender not listed here ____

○ Prefer not to answer

What is your race and ethnicity? Please select all that apply.

- ☐ American Indian, Alaska Native, First Nations
- ☐ Asian, Asian American
- ☐ Black, African American
- ☐ Hispanic
- ☐ Latino/a/e
- ☐ Middle Eastern, North African, Arab American
- ☐ Native Hawaiian, Pacific Islander
- ☐ White, Caucasian
- ☐ A race or ethnicity not listed here

What is your sexual orientation?

- ○ Bisexual
- ○ Gay or lesbian
- ○ Heterosexual or straight
- ○ Prefer not to answer
- ○ An orientation not listed here____

In which country do you currently live?

____

In which city, state/providence/territory do you currently live? *This is required for survey validation.

____

Please indicate your parent pregnancy loss experience.

○ I am the person who was pregnant when there was a loss.

○ I am the person who had a surrogate pregnancy when there was a loss.

○ I am the partner of someone who was pregnant when there was a loss.

○ I am the person who was the intended parent of a surrogate pregnancy when there was a loss.

○ I had an experience not listed here ____

Please confirm the option closest to your experience as a loss parent. *This is required for survey validation.

○ I am the person who was pregnant when there was a loss.

○ I am the person who had a surrogate pregnancy when there was a loss.

○ I am the partner of someone who was pregnant when there was a loss.

○ I am the person who was the intended parent of a surrogate pregnancy when there was a loss.

○ I had an experience not listed here ____

How many pregnancies have you or your partner experienced?

Please indicate the number of pregnancies you or your partner experienced using only the format that is highlighted (numbers, words). *This is required for survey validation.

How many pregnancy losses have your or your partner experienced?

What type of loss(es) have you or your partner experienced? Please select all that apply.

- ☐ Neonatal death
- ☐ Stillbirth
- ☐ I experienced a loss not listed here (e.g., miscarriage, termination for medical reasons)

How many stillbirths have you experienced?

- ○ 0
- ○ 1
- ○ 2
- ○ 3
- ○ 4
- ○ 5+

In what year did your loss occur? Please use the format XXXX.

What was your baby's approximate gestational age in weeks? Please use the format XX.

Traditionally, researchers give alternative names to participants, specific locations, etc. to maintain confidentiality of the participants. However, we recognize the desire of some parents to keep the memory of their child(ren) going by sharing their name. Please let us know how you would like us to use your baby's name(s). If left blank, we will keep confidentiality of your baby's name.

- ◯ I would like you to use my baby's real name.
- ◯ I would like you to use an alternative name.

What did the healthcare professionals (e.g., doctors, nurses) do or offer that was helpful or provided comfort for you while at the hospital? Please select all that apply.

	Did / Offered	Was Helpful
Offered guidance / recommendations on making memories	☐	☐
Encouraged making memories (e.g., holding baby, taking photos)	☐	☐
Offered to clip / clipped baby's hair	☐	☐
Offered to create / created hand or foot molds	☐	☐
Offered to create / created hand or foot prints	☐	☐
Offered to take / took photographs	☐	☐
Asked baby's name	☐	☐
Said baby's name	☐	☐

Complimented your baby (e.g., she's perfect, he's handsome)	☐	☐
Expressed condolences	☐	☐
Put a sign on the door indicating pregnancy loss	☐	☐
Located room in a place separate from labor and delivery rooms with live births	☐	☐
Told us what happened was not our fault	☐	☐
Provided information on baby's appearance	☐	☐
Gave resources about pregnancy loss and/or grief	☐	☐
Recommended counseling/therapy services	☐	☐
Provided information about funeral/cremation services	☐	☐
Provided religious personnel/resources	☐	☐
Provided social work services	☐	☐
Other __	☐	☐
Other __	☐	☐
Other __	☐	☐

While at the hospital, did you have support from someone who had experienced a loss similar to yours?

- ◯ Yes, a personal connection
- ◯ Yes, a healthcare professional
- ◯ Yes, an organization
- ◯ No, I did not have support from someone who had experienced this type of loss

Before your baby was born, did you receive any educational materials from healthcare professionals **about stillbirth**?

- ◯ Yes
- ◯ No
- ◯ I do not remember

Before your baby was born, did you receive any educational materials from healthcare professionals about **grief related to pregnancy or infant loss**?

- ◯ Yes
- ◯ No
- ◯ I do not remember

Did you or your partner make memories with your baby at the hospital?

- ◯ Yes
- ◯ No

What activities did you or your partner do at the hospital? Please select all that apply.

	Me	My partner
Bathe your baby	☐	☐
Clip / ask for clippings of hair	☐	☐
Create / ask for hand or foot molds	☐	☐
Create / ask for hand or foot prints	☐	☐
Dress your baby	☐	☐
Name your baby	☐	☐
Read to your baby	☐	☐
Sing to your baby	☐	☐
Spend time with your baby	☐	☐
Take photos / ask for photos of your baby	☐	☐
Talk to your baby	☐	☐
Other __	☐	☐
Other __	☐	☐
Other __	☐	☐

We make the best decisions we can in the moment, but looking back, is there anything you would do differently while at the hospital before, during, or after the birth of your baby?
