# Supplementary material for: Parent hospital experiences following stillbirth
Source: Front Psychiatry. 2026 Feb 4;17:1706931. doi: 10.3389/fpsyt.2026.1706931 (PMC12914944; doi:10.3389/fpsyt.2026.1706931)
Supplement: Supplementary file 2 [file DataSheet2.pdf]

Appendix A

Summary of other memory making activities parents did at the hospital

Theme	n	Examples
Physical contact memory making	18	"Kissed and held him"<br>"Slept with her in my arms"<br>"Wear Emma in my baby wrap"
Religious memory making	10	"Baptized my baby"<br>"Just held him and prayed for him" "Priest came"
Family memory making	10	"Had close family visit"<br>"Had her siblings come to see her"<br>"We had all family members who wanted to meet Tristan come to meet him"
Unique activities of memory making	8	"Changed diaper"<br>"Danced"<br>"Took baby for a walk outside"
Physical memento memory making	6	"Made bracelets"<br>"He was given a stuffed animal that we took pictures with and were allowed to keep"

Note. n = 40

Appendix B

Summary of "other" responses for what healthcare professionals did or offered that was helpful for provided comfort while at the hospital

Theme	n	Examples
Tangible support	35	"Made a memorial box for him at the hospital"
		"Offered a cuddle cot so baby could stay in the room longer"
		"Let us pick a hat and blanket for her" "Allowed me to be with my baby as long as I wanted"
Emotional support	19	"Grieved with us"
		"Sent a note after expressing condolences"
		"Helped my parents and children be allowed to come in outside of visiting hours"
Informational support	13	"Offered guidance on options with birth and options after…"
		"Provided lactation support/info"
		"Told us about the Share program at [hospital]"
Trained professional support	5	"Had a specified bereavement team" "Grief doula was arranged that did all of the above"

Note. $n = 48$
